# Changes in the autonomic cardiorespiratory activity in parturient women with severe and moderate features of preeclampsia

**DOI:** 10.3389/fimmu.2023.1190699

**Published:** 2023-09-01

**Authors:** Elias Yojairi Pichardo-Carmona, José Javier Reyes-Lagos, Rosselin Gabriela Ceballos-Juárez, Claudia Ivette Ledesma-Ramírez, Hugo Mendieta-Zerón, Miguel Ángel Peña-Castillo, Ejay Nsugbe, Miguel Ángel Porta-García, Yecid Mina-Paz

**Affiliations:** ^1^ School of Medicine, Autonomous University of the State of Mexico (UAEMéx), Toluca, Mexico; ^2^ Mónica Pretelini Sáenz Maternal-Perinatal Hospital, Health Institute of the State of Mexico (ISEM), Toluca, Mexico; ^3^ Basic Sciences and Engineering Division, Metropolitan Autonomous University (UAM-I), Iztapalapa, Mexico; ^4^ Nsugbe Research Labs, Swindon, United Kingdom; ^5^ Faculty of Health Sciences, Universidad Libre Seccional Cali, Cali, Colombia

**Keywords:** vagal withdrawal, mutual information, pregnancy, cardiorespiratory coupling, pulse-respiration quotient

## Abstract

**Background:**

Cardiorespiratory coupling (CRC) is a physiological phenomenon that reflects the mutual interaction between the cardiac and respiratory control systems. It is mainly associated with efferent vagal activity from the central autonomic network. Few studies have explored the autonomic changes of CRC in preeclampsia, a critical obstetric complication related to possible autonomic dysfunctions and inflammatory disturbances. This study examined the autonomic mechanisms of CRC in women with severe and moderate preeclampsia and healthy controls by applying nonlinear methods based on information theory, such as mutual information (MI) and Renyi’s mutual information (RMI) and the linear and nonlinear analysis of the Pulse-Respiration Quotient (PRQ).

**Methods:**

We studied three groups of parturient women in the third trimester of pregnancy with a clinical diagnosis of preeclampsia without severe symptoms (P, 38.5 ± 1.4 weeks of pregnancy, n=19), preeclampsia with severe symptoms (SP, 37.5 ± 0.9 weeks of pregnancy, n=22), and normotensive control women (C, 39.1 ± 1.3 weeks of pregnancy, n=20). 10-minutes of abdominal electrocardiograms (ECG) and respiratory signals (RESP) were recorded in all the participants. Subsequently, we obtained the maternal beat-to-beat (RR) and breath-to-breath (BB) time series from ECG and RESP, respectively. The CRC between RR and BB was quantified by nonlinear methods based on information theory, such as MI and RMI, along with the analysis of the novel index of PRQ. Subsequently, we computed the mean PRQ (mPRQ) and the normalized permutation entropy (nPermEn_PRQ) from the PRQ time series generated from BB and RR. In addition, we examined the vagal activity in the three groups by the logarithm of the median of the distribution of the absolute values of successive RR differences (logRSA).

**Results:**

The MI and RMI values were significantly lower (p<0.05) in the preeclamptic groups compared to the control group. However, no significant differences were found between the preeclamptic groups. The logRSA and nPermEn_PRQ indices were significantly lower (p<0.05) in SP compared to C and P.

**Conclusion:**

Our data suggest that parturient women with severe and mild preeclampsia may manifest an altered cardiorespiratory coupling compared with normotensive control women. Disrupted CRC in severe preeclampsia could be associated with vagal withdrawal and less complex cardiorespiratory dynamics. The difference in vagal activity between the preeclamptic groups may suggest a further reduction in vagal activity associated with the severity of the disease.

## 1 Introduction

A well-known feature of oscillatory and biological oscillators is synchronization; heart and respiratory rates are no exception. Previous studies of biological rhythms conclude the existence of reciprocal interaction between these two physiological rhythms or between the cardiac autonomic and respiratory control systems, called the cardio-respiratory coupling or CRC (1). Particularly, relevant studies have mainly focused on heart rate variability (HRV) to evaluate cardiac autonomic activity in women with preeclampsia, suggesting that preeclamptic women may show increased cardiac sympathetic activity with decreased parasympathetic activity compared to normotensive control women (2–5). Novel evidence indicates that changes to the central respiratory network’s connections with the sympathetic nervous system and the vagus nerve may significantly impact CRC (6). However, the autonomic mechanisms involved in preeclampsia have not yet been fully explored by quantifying the CRC. According to relevant findings, assessing the CRC can provide synergies that support healthy physiology and offer a wide range of potential advantages (7). Additional evidence supports that CRC is mediated mainly within the central nervous system, with relevant vagal influence (8).

Few studies have simultaneously explored multivariate physiological interactions in preeclampsia or labor. For example, a relevant study examined the linkage between breathing and the cardiovascular system to better understand the dynamic alterations brought on by preeclampsia. The authors reported that the nonlinear form of the respiratory influence on the heart rate significantly differs between healthy and pathological groups (9). In addition, other studies have found increased cardiorespiratory coordination in preeclamptic women; it is considered a particular type of CRC. The authors considered that high cardiorespiratory coordination could be accompanied by sympathicotonia (10).

In addition to the fact that the literature reports few cases of evaluation of CRC in preeclampsia, the severity features of preeclampsia must also be considered a key factor of autonomic regulation. Specifically, Lakhno reported that gradual alterations of sympathovagal balance were associated with the progressing severity of preeclampsia (3). Furthermore, a recent study of our research group suggests that diminished parasympathetic activity may be exhibited in preeclamptic women during nonlabor/labor compared to normotensive healthy women (11).

Nonlinear methods based on information theory and the dynamic complexity of CRC have not yet been entirely applied in the obstetrics area. Mainly, oscillator coupling is widely applicable in both physical and biological systems. With the help of standard techniques for time-series analysis of information theory, interactions between oscillators can be empirically studied (12). As an alternative to the conventionally linear correlation analysis, the mutual information (MI) measures give access to nonlinear cardiorespiratory interdependencies (13), and it can quantify the CRC (14). Noteworthy, MI is widely acknowledged as a powerful technique for measuring nonlinear relationships between two variables (14); it quantifies the information that can be acquired about a random variable through another variable. Furthermore, MI possesses a calculation advantage by being a model-free approach, allowing it to be sensitive to various types of autonomic interactions (15).

Promising evidence about the assessment of CRC as a relevant physiological mechanism has been reported in mental arithmetic and sustained attention (16), emotion characterization (17), and lipopolysaccharide-induced endotoxemia (18). In addition, the neuroinflammatory state of preeclampsia may be related to changes in nonlinear features of physiological signals (19).

Various techniques exist for exploring CRC, among which the pulse respiration quotient (PRQ) has been recognized as a highly effective and relevant tool for obtaining valuable information on autonomic nervous system regulation patterns (20). The PRQ is a valuable metric that captures a distinct and singular facet of cardiorespiratory activity; it is calculated by dividing the heart rate by the respiration rate. The PRQ is an intriguing index with great potential in pathophysiology and human physiology research. Although it has received less attention, the PRQ has been recognized as a valuable tool for assessing cardiorespiratory activity and providing insightful information (21). Studies have revealed that certain medical conditions, including myocardial infarction and hyperthyroidism, exhibit an elevated PRQ and diminished circadian variation (21). Existing research supports the notion that the exploration of adaptability in CRC can be significantly enhanced by precisely calculating diverse indices computed from PRQ time series. In healthy individuals, the regulation of cardiorespiratory functions exhibits a notable level of autonomic adaptability, particularly in response to posture and breathing patterns (22). According to the consulted literature, no studies are available that quantify the CRC changes by applying the PRQ and information theory methods in parturient preeclamptic women.

This study explored the autonomic mechanisms of cardiorespiratory coupling in women with severe and moderate preeclampsia and healthy controls by applying nonlinear methods based on information theory, such as mutual information and entropy, and quantifying the PRQ time series. Assuming that preeclamptic women are a suitable model of high sympathetic, low parasympathetic activity, reduced heart rate complexity, and exacerbated inflammation (19), these neuroimmune alterations may be reflected in linear and nonlinear features of the maternal cardiorespiratory coupling.

## 2 Methods

### 2.1 Participants

Following a cross-sectional case-control study design, we studied parturient Mexican women aged between 18 and 35 years in their third trimester of pregnancy (36–40 weeks of gestation). The study was conducted at the Emergency and Obstetrical Surgery Department of the Maternal-Perinatal Hospital “Mónica Pretelini Sáenz” in Toluca de Lerdo, Mexico State, Mexico, from February 2021 to October 2022. Before recording physiological measurements, all participants were informed about the study’s purpose, and informed consent was obtained from those who voluntarily participated. The study protocol received approval from the institution’s research ethics committee (registration number: 2021-03-719) and adhered to all relevant institutional and general ethical guidelines throughout its execution.

Participants were classified into three groups: preeclampsia without features of severity or mild preeclampsia (P, n=19), preeclampsia with features of severity or severe preeclampsia (SP, n=22), and normotensive control women (C, n=20).

The severity of preeclampsia in the participants was diagnosed by medical doctors at the Maternal-Perinatal Hospital, following the Clinical Management Guidelines for Obstetricians-Gynecologists of the American College of Obstetricians and Gynecologists (ACOG) and the clinical practice guide of the Mexican Social Security Institute (IMSS) (23, 24). Preeclampsia without severe features (P) was confirmed if the systolic blood pressure measured 140 mm Hg or more or the diastolic blood pressure measured 90 mm Hg or more on two occasions at least 4 hours apart. The presence of proteinuria (urine protein-creatinine ratio or UPCR greater than 0.28) (24) was the specific biochemical marker that helped to differentiate preeclampsia from gestational hypertension (25). On the other hand, preeclampsia with severe features (SP) was confirmed if the systolic blood pressure was measured at 160 mm Hg or more or if the diastolic blood pressure was 110 mm Hg or more on two occasions at least 4 hours apart.

Parturient women during the latent and active phases of labor were included in the study. Exclusion criteria for all groups include the presence of chronic or gestational hypertension, diabetes mellitus, autoimmune, renal, or cardiovascular disease, and administration of epidural blockade during parturition. Participants’ clinical data, such as age, weeks of pregnancy, stage of labor, and temperature, were also gathered before recordings. Participants were placed on a controlled diet during the assessment, which allowed them to consume water. Additionally, due to their pregnancy condition and following hospital guidelines, they were advised to abstain from consuming caffeine or any substances that could potentially affect the fetus during pregnancy.

### 2.2 Data acquisition and preprocessing

**Figure 1:**
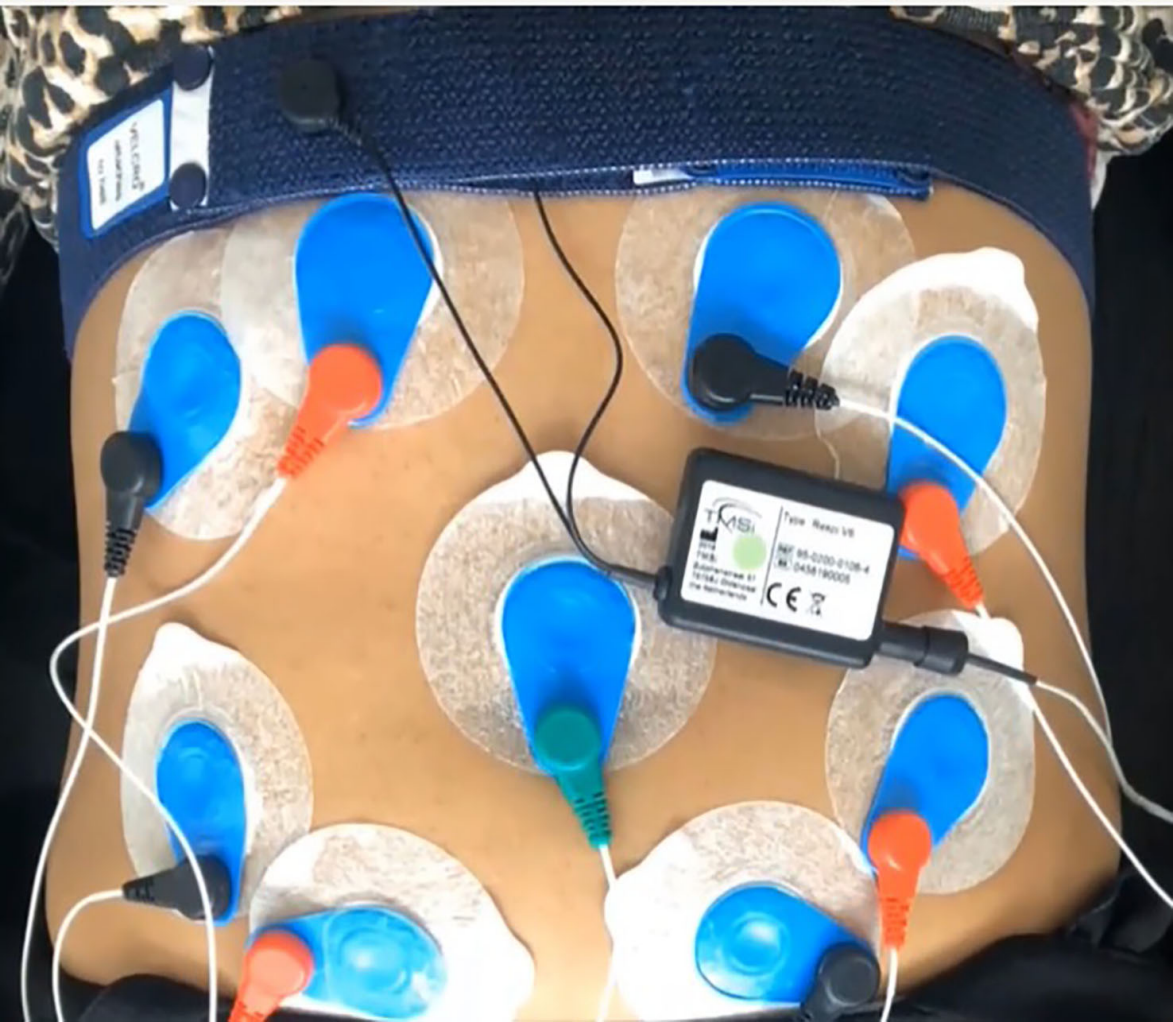
Experimental setup of data acquisition for maternal abdominal electrocardiograms (ECG) and respiratory signals (RESP).

We used a portable data acquisition device (Mobi mobile amplifier system, TMSi Systems, The Netherlands) with four channels of bipolar electrode position on the maternal abdomen and a respiration sensor attached to a belt-type strap on the chest. The respiratory transducer (respiration effort module V6, Mobi, TMSi Systems, The Netherlands) is a rugged strain assembly that measures maternal thoracic or abdominal circumference changes. 10-minutes of maternal abdominal electrocardiogram (ECG) and respiratory signals (RESP) were recorded in all the participants in a semi-fowler position with a sampling frequency of 1000 Hz (**Figure 1**). All recordings were performed between 08:00 am to 12:00 pm to minimize diurnal variations in the physiological parameters. The physiological measurements were also recorded in a private cubicle within the Emergency and Obstetrical Surgery Department at a controlled temperature of approximately 15°C with air conditioning and under standard hospital illumination provided by fluorescent light sources.

We obtained the maternal beat-to-beat (RR) and breath-to-breath (BB) time series from ECG and RESP signals, calculated as the distance between consecutive R-peak to R-peak and breath-peak to breath-peak intervals on the ECG and RESP signals, respectively. The R-peak detection by the Pan-Tompkins method was performed to preprocess one maternal abdominal ECG channel. The initial step of the Pan-Tompkins filter involved applying a bandpass filter (5-15 Hz) to the data to enhance the low signal-to-noise ratio (26). Analogously, the RESP signals were digitally filtered with a bandpass filter with cutoff frequencies between 0.2 to 0.5 Hz. These cutoff frequencies were determined considering a normal range of respiratory rate (12 to 30 breaths per minute) (27). It is important to mention that the chosen bandwidth for filtering was based on previous studies (28–30). In order to remove any ectopic beat or respiration from the maternal short-term RR and BB interval time series obtained, adaptive filtering was applied to both time series as a preprocessing step (31). The maternal RR and BB time series were interpolated with a cubic spline at 4 Hz to re-construct uniformly sampled versions of the RR and BB time series and make both time series equidistant. Then, the DC trend was removed from each RR and BB signal by subtracting its mean value. Finally, both signals were normalized by dividing the data by the maximum RR or BB value. All computations were made using Matlab software (The MathWorks Inc., Massachusetts, United States).

### 2.3 CRC assessment by mutual information and Renyi’s mutual information

The quantitative data analysis for CRC assessment was performed using the following nonlinear methods based on information theory: mutual information (MI) and Renyi’s mutual information (RMI) between the normalized and interpolated maternal RR and BB time series. These algorithms are freely available scripts for Matlab in the MIToolbox software (32).

Mutual information (MI) measures two signals’ nonlinear dependence, allowing to detection of shared information between two variables. It is computed based on the distributions of values within variables and the joint distribution of two or more variables (33). It may be used to explore the propagation of information between two-time series. It is a versatile framework that may be used for linear, nonlinear, or circular data distributions. MI is the amount of shared information between two variables, obtained from the sum of individual entropies of these two variables and subtracting their joint entropy (34). More formally, MI is defined as:


Equation 1 MI(X,Y)=H(X)+H(Y)−H(X,Y)

Where H  represents the Shannon entropy and joint entropy of time series X and Y, respectively.

On the other hand, Renyi’s mutual information $RMI_{\alpha }\left(X,Y\right)$ is defined as the Renyi divergence between joint distributions of X  and Y  and the product of its marginal distributions ${\text{P}}_{XY}$ , ${\text{P}}_{X}{\text{P}}_{Y}$ , i.e.:


Equation 2 $$RMI_{\alpha }\left(X,Y\right)=D_{\alpha }\left({\text{P}}_{XY}||{\text{P}}_{X}{\text{P}}_{Y}\right)$$

where α is used to find the divergence between two probability distributions, it is defined ∀ α ≠ 1. RMI depends on the α value; in this study, we explored α=2.5 (18). The $D_{\alpha }\left({\text{P}}_{XY}||{\text{P}}_{X}{\text{P}}_{Y}\right)$ denotes the Kullback Leibler divergence (KL divergence) between the joint probability ${\text{P}}_{XY}$ and the product ${\text{P}}_{X}{\text{P}}_{Y}$ of the probability distribution of X and Y. Large MI and RMI values imply heavily dependent time series, while lower values indicate nearly independent time series (35). Thus, higher values of MI and RMI between the maternal RR and BB time series may be associated with the manifestation of CRC. The main difference between Mutual Information and Renyi Mutual Information lies in their mathematical formulation and how they capture dependence. MI measures overall dependence, while RMI provides a flexible parameter to adjust the sensitivity to different dependence patterns. Measuring both indices is crucial as they offer complementary information, allowing a more comprehensive assessment of the dependence of data (36).

### 2.4 Vagal activity assessment

We estimated the vagal activity from the RR time series using the reliable time-domain approach known as the logarithm of the median of the distribution of the absolute values of successive differences (logRSA). Topçu et al. (37) demonstrated the relationship between this approach and cardiorespiratory interactions. The following equation was applied to compute the logRSA:


Equation 3 $$logRSA=log\left[median\left|RR_{i+1}-RR_{i}\right|\right]$$

where RR are the interbeat intervals that follow one another, and the median value is calculated throughout the absolute value ( $\left|RR_{i+1}-RR_{i}\right|$ ) of the successive differences of the RR time series. Unlike other linear indices of HRV, the logRSA has demonstrated robustness and the ability to differentiate between vagal states (37).

### 2.5 Analysis of maternal pulse-respiration quotient (PRQ) time series

As a quantitative marker to evaluate linear and nonlinear features of CRC, we introduced the Pulse-Respiration Quotient (PRQ) time series analysis to explore potential changes in the cardiorespiratory dynamics between groups. Two methods are available to determine the PRQ; RR : BB = m:1. The first approach involves a manual technique where the pulse is palpated or visually observed (38), and the number of heartbeats corresponding to each breathing cycle is counted. However, this method lacks the precision required for modern scientific evaluations. The second approach involves recording both the ECG and breathing signals, automatically calculating RR intervals and BB time series intervals, and calculating the ratio of RR to BB. Furthermore, if RR and BB are expressed as counts per minute, the instantaneous heart rate (HR) and breathing rate (BR) can be obtained using the following formulas:


Equation 4 $$\;Heart\;rate\;(HR)=\frac{60}{RR}\;\left[\text{heartbeats}/\text{min}\right]$$

Equation 5 $$Breathing\;rate\;\left(BR\right)=\frac{60}{BB}\;\left[\text{respirations}/\text{min}\right]$$

The PRQ analysis captures a specific and unique element of the cardiorespiratory activity. In our case, the interpolated maternal RR and BB time series from all groups were transformed into instantaneous HR (Equation 4) and BR (Equation 5) times series to calculate the PRQ time series. Consequently, the instantaneous PRQ was computed by dividing the instantaneous HR by BB:


Equation 6 $$PRQ=\frac{HR}{BR}$$

According to Scholkmann and Wolf, it is crucial to deviate from the conventional method of determining the PRQ by simply averaging heart rate and breathing rate measurements. Instead, it is imperative to employ an algorithm to continuously calculate heartbeat intervals for each respiratory cycle in real-time when determining the PRQ (21). We used the interpolated and equidistant BB and HR time series to compute the PRQ time series. Thus, the PRQ time series were calculated as the ratio of instantaneous heart rate (HR) to instantaneous breathing rate (BR) or as the ratio of HR to BR (Equation 6) recorded over 10 minutes (21).   Here, the functional PRQ was explored. Interestingly, the state in which the PRQ hovers around 4, known as “PRQ normalization” represents an optimal PRQ value concerning cardiovascular system function. This term emphasizes the importance of this state in human physiology (21).

The PRQ time series were analyzed using linear and nonlinear indices in the Pybios software (39), such as the mean value of the PRQ time series (mPRQ), sample entropy with *r=0.15* and *m*=2 (40) (SampEn_PRQ) and the normalized permutation entropy of PRQ time series (nPermEn_PRQ) with *m*=3 (41). Lower entropy values reflect more regularity and predictability, while higher ones manifest more unpredictability (42, 43). The selection of specific indices for analyzing PRQ time series is based on their relevance and advantages in capturing relevant information. The mPRQ provides a summary measure of overall PRQ levels (21). Additionally, entropic measures have been widely used and validated in previous studies for characterizing physiological dynamics and assessing the regulatory mechanisms underlying cardiorespiratory interactions (44, 45).

### 2.6 Statistical analysis

We performed statistical comparisons between the three groups (C, P, and SP) for the mean values of MI, RMI, logRSA, PRQ indices, and clinical characteristics of participants. The normality was examined using Shapiro-Wilks because the sample size was less than 50 participants. For data that followed a normal distribution, parametric statistical analysis methods (one-way ANOVA) were applied, followed by uncorrected Fisher's LSD for post-hoc multiple comparisons. Additionally, we calculated the effect size using Cohen's f to measure the average effect across the three groups. The effect size analysis was conducted using G*Power software (Universität Düsseldorf, Düsseldorf, Germany). The following comparisons were conducted to assess the differences: C vs. P (Control vs. Preeclampsia), C vs. SP (Control vs. Severe Preeclampsia), and P vs. SP (Preeclampsia vs. Severe Preeclampsia). All tests were performed with a significance level of α = 0.05. All the results were analyzed using statistical tests with GraphPad Prism 8.0 (GraphPad Software, Inc., La Jolla, CA, USA).

## 3 Results

**Table 1** depicts the relevant clinical characteristics of the three studied groups. Data are shown as mean ± SD. We found statistical differences in some clinical characteristics that confirmed the medical diagnosis of preeclampsia. For example, the ANOVA results indicated significant differences among the groups for changes in systolic blood pressure (F=41.6, p<0.0001, Cohen’s f=1.13). *Post-hoc* multiple comparisons revealed that the preeclampsia without severe features (P) group had significantly higher systolic blood pressure compared to the Control group or C (p<0.0001). Furthermore, the preeclampsia with severe features (SP) group exhibited even higher systolic blood pressure than the Control group (p<0.0001).

**Table 1:** Mean (± SD) of the clinical characteristics of participants: normotensive or control (C), preeclampsia without severe features (P), and preeclampsia with severe features (SP).

Clinical characteristics	C (n=20)	P (n=19)	SP (n=22)
Age (years)	24.4 ± 5.2	26.5 ± 5.4	27.5 ± 7.1
Gestational age (weeks)	39.1 ± 1.3	38.5 ± 1.4#	37.5 ± 0.9*
BMI (kg/m^2^)	29.6 ± 5.6	31.8 ± 4.9	29.5 ± 8.3
Systolic blood pressure (mm/Hg)	117.9 ± 11.9	140.9 ± 8.8+	152.9 ± 15.5*
Diastolic blood pressure (mm/Hg)	73.6 ± 9.6	90.7 ± 6.7+,#	101.1 ± 10.3*
Urine Protein Creatinine Ratio or UPCR (mg/mg)	–	0.9 ± 1.1#	1.4 ± 1.6
Mean Heart Rate (bpm)	79.2 ± 12.8	81.2 ± 16.9#	94.5 ± 14.4*
Mean Breathing Rate (rpm)	22 ± 4	21 ± 3	22 ± 4
Temperature (°C)	36.4 ± 0.3	36.8 ± 0.8	36.3 ± 0.3
Latent phase of labor (%)	100%	90%	90%
Active phase of labor (%)	0%	10%	10%
C-section (%)	80%	80%	90%

*p<0.05 between C and SP.

+p<0.05 between C and P.

#p<0.05 between P and SP.

Similarly, results indicated significant differences among the groups for changes in diastolic blood pressure (F=48.7, p<0.0001, Cohen’s f=1.23). *Post-hoc* multiple comparisons revealed that the diastolic blood pressure was significantly higher in the SP group than in the Control and P groups (p<0.0001). Furthermore, the severity of preeclampsia was associated with a significant increase in Urine Protein Creatinine Ratio or UPCR, with the SP group showing higher values than the P group (p<0.05).

**Figure 2:**
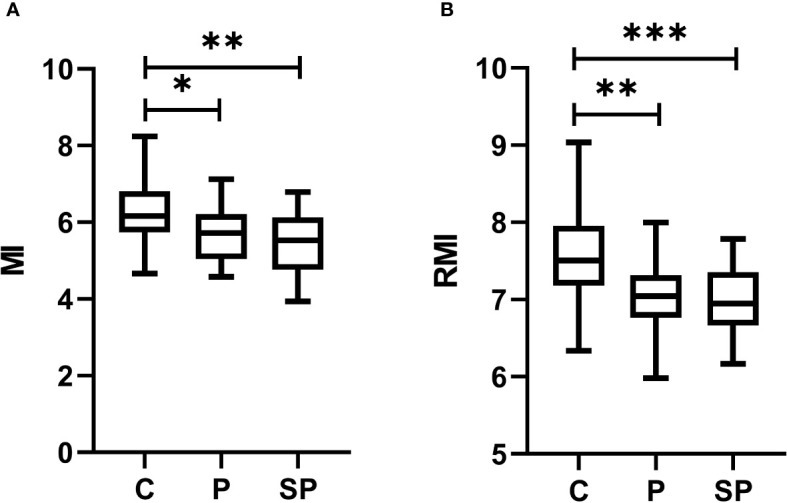
Box plots of nonlinear methods based on information theory to evaluate the cardiorespiratory coupling (CRC) among healthy normotensive participants (C), preeclampsia without severe features (P), and preeclampsia with severe features (SP) groups. **(A)** mutual information (MI); **(B)** Renyi’s mutual information with a degree of divergence α = 2.5 (RMI); *p< 0.05, **p<0.01,***p<0.001 indicate significant differences by *post-hoc* LSD Fisher.

The ANOVA results indicated significant differences among the groups for changes in mutual information (MI) (F=5.73, p=0.0053, Cohen’s f=0.42). *Post-hoc* multiple comparisons revealed that MI showed significant differences between the control and the preeclamptic groups, with P and SP having a lower MI than the C group (**Figure 2A**, p<0.05). Similarly to MI, results indicated significant differences among the groups for changes in RMI (F=7.44, p=0.0013, Cohen’s f=0.48). It showed significant differences between the control and preeclamptic groups, with the P group having a lower RMI than the C group (**Figure 2B**, p<0.01). In addition, the RMI values were significantly lower in the SP group compared to the Control group (**Figure 2B**, p<0.001).

**Figure 3:**
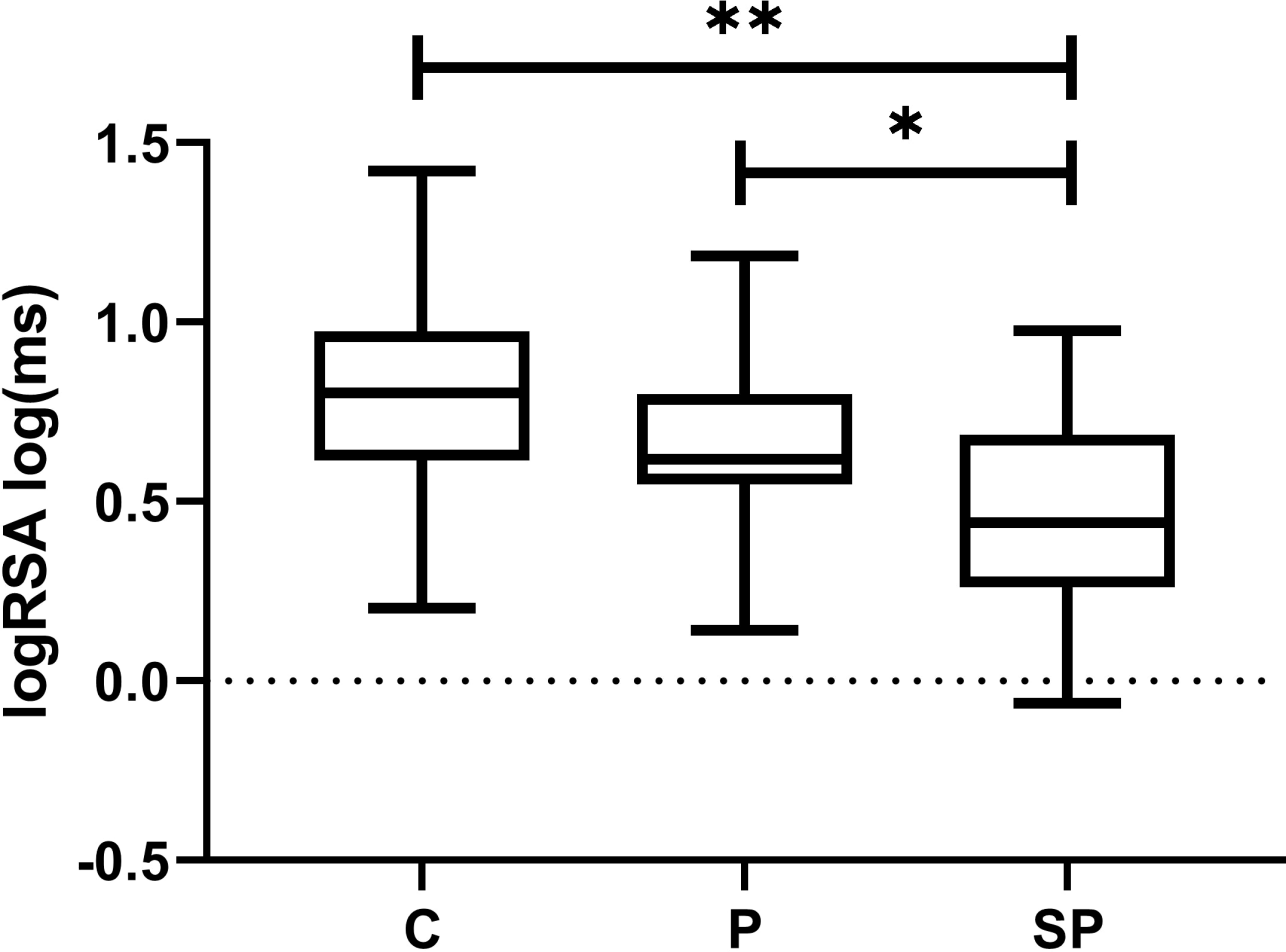
Box plots of the vagal-related index of the logarithm of the median of the distribution of the absolute values of successive RR differences (logRSA) for normotensive participants (C), preeclampsia without severe features (P), and preeclampsia with severe features (SP) groups. *p<0.05 indicates significant differences between P vs. SP by *post-hoc* LSD Fisher; **p<0.01 indicates significant differences between C vs. SP by *post-hoc* LSD Fisher.

We found significant differences in the vagal activity, indicated by the logRSA index (F=7.44, p<0.01, Cohen’s f=0.46), between the Control and SP groups, with the Control group having a higher logRSA index than the SP group (**Figure 3**, p<0.01). Interestingly, we also found statistical differences between the P and SP groups in vagal activity, with the P group having a higher logRSA index than the SP group (**Figure 3**, p<0.05). For comparison purposes, we also calculated other relevant indices of vagal activity, such as the high frequency (HF) of heart rate variability and the root mean square of successive differences (RMSSD). However, no significant differences were found (data not shown).

**Figure 4:**
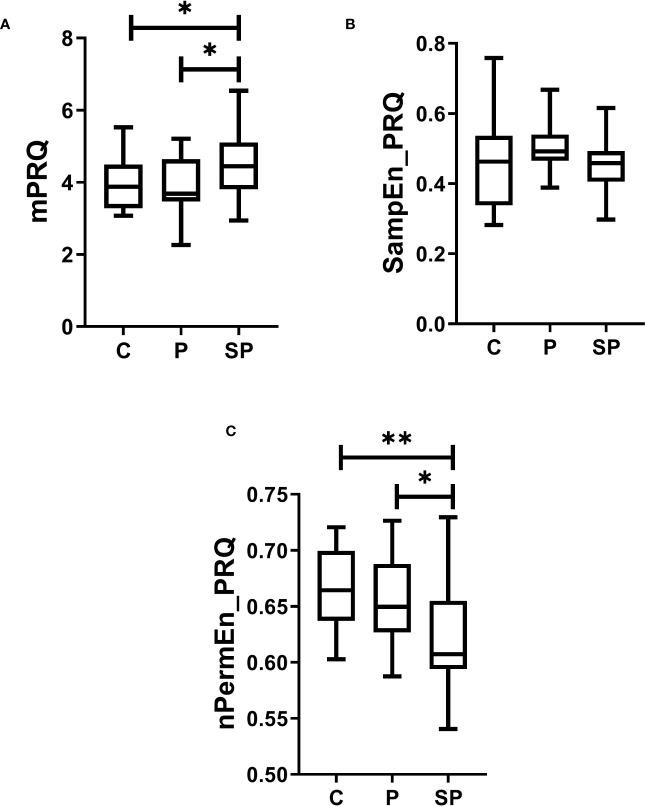
Box plots of linear and nonlinear analysis of maternal Pulse-Respiration Quotient (PRQ) time series for normotensive participants (C), preeclampsia without severe features (P), and preeclampsia with severe features (SP) groups. **(A)** the mean value of the PRQ time series (mPRQ), **(B)** sample entropy (SampEn_PRQ), and **(C)** the normalized permutation entropy (nPermEn_PRQ). All these indices were calculated for the PRQ time series. *p<0.05 and **p<0.01 indicate significant differences by *post-hoc* LSD Fisher.

Finally, the results of the ANOVA revealed significant differences among the groups in terms of changes in mPRQ (F=3.02, p<0.05, Cohen’s f=0.30). *Post-hoc* multiple comparisons demonstrated statistical differences in mPRQ, with the SP group showing significantly higher values than the Control and P groups (**Figure 4A**, p<0.05). Additionally, nPermEn_PRQ exhibited significant differences (F=5.46, p<0.01, Cohen’s f=0.41); it showed statistical differences between the Control and preeclamptic groups, with the Control group having a higher nPermEn_PRQ value than the SP and P groups (**Figure 4C**, p<0.05). Both mPRQ and nPermEn_PRQ exhibited significant differences between P and SP (p<0.05). Furthermore, there were no significant differences in SampEn_PRQ (**Figure 4B**).

## 4 Discussion

Our exploratory study presents novel evidence on cardiac and respiratory interactions quantifying the CRC by nonlinear methods based on information theory and assessing PRQ time series in parturient women with a clinical diagnosis of preeclampsia. Our preliminary findings indicate that parturient women with severe and mild features of preeclampsia may have altered CRC than normotensive women. In severe and mild preeclampsia, lower CRC (indicated by decreased values of MI and RMI than in control women; **Figure 2**) may be related to autonomic modifications. Remarkably, these autonomic changes in the CRC introduced particularly by preeclampsia with severe features could be attributed to diminished vagal activity (indicated by lower values of logRSA). In addition, preeclampsia with severe features may be associated with less complex cardiorespiratory dynamics (indicated by decreased values of nPermEn_PRQ than in normotensive women; **Figure 4C**). Such observations have not been reported by others previously, to our knowledge.

The CRC is known to be diminished when there is a shift in the balance between sympathetic and vagal activity, with a prevalence of sympathetic activity and vagal withdrawal, as demonstrated in a relevant study (46). This observation remains consistent even in trained individuals (47). The vagal activity, indicated by the logRSA index, was found to have significant differences between C and SP and between the P group and SP. It suggests that severe preeclampsia could be associated with lower vagal activity than the normotensive and mild preeclampsia groups. The subtle difference in vagal activity between mild preeclampsia and severe preeclampsia group may suggest a further reduction in vagal activity with the severity of the disease.

This work stands out from relevant physiological signal processing studies of preeclampsia that only use univariate physiological measures to detect cardiac autonomic changes by maternal heart rate variability analysis (4, 48, 49, 2, 50). The main findings of these relevant previous works have reported increased cardiac sympathetic activity in preeclampsia, or a state of sympathetic hyperactivity with decreased parasympathetic control of heart rate. This pertinent evidence aligns with our present findings, given that the mean heart rate in SP and P groups was significantly higher than controls (**Table 1**). A previous study of our research group showed that lower cardiac parasympathetic response might be manifested in preeclamptic women during labor/nonlabor compared to normotensive women (11).

The mother’s heart rate rises during the initial weeks of pregnancy and reaches its highest point in the late second to early third trimester (51). Therefore, by the third trimester of pregnancy, the changes in the maternal heart rate should have already stabilized in our study groups. Patients with preeclampsia have been found to exhibit increased sympathetic function, leading to higher excitability of peripheral vascular resistance (52, 53). Schobel et al. (54) utilized muscle sympathetic nerve recording and suggested that preeclampsia is characterized by sympathetic overactivity, which tends to return to normal levels after delivery. Øian et al. (55) also reported a significant increase in arterial epinephrine levels in the preeclamptic group, correlating with mean arterial pressure and heart rate. On the other hand, findings from previous studies have indicated a higher heart rate and reduced heart rate variability during a deep breathing test, suggesting a potential impairment of the vagal reflex in preeclampsia (56).

In addition, increased mean PRQ value may be associated with CRC changes in the SP group. According to Scholkmann & Wolf, 2019, a PRQ = 4 suggests a coupling between the respiratory and cardiac systems. We found a mean PRQ closer to 4 in the control group and mild preeclampsia (higher CRC) than in the SP group (lower CRC) (**Figure 4A**). In addition, lower nPermEn_PRQ values may reflect more regularity and predictability (less complexity in the cardiorespiratory system) in the severe preeclampsia group than in controls and mild preeclampsia. Our results are consistent with evidence that patients with acute myocardial infarction and hyperthyroidism have higher PRQ values (compared to healthy controls) (57). Additionally, persons with autonomic dysfunctions exhibit increased PRQ values when changing from supine to standing compared to healthy controls (21), supporting our previously mentioned results.

The MI and RMI indices quantify the information shared between two variables. Both indices assign a high value when the study variables are strongly correlated and a low value when they are strongly independent. We consider that MI and RMI indices applied to assess the CRC in preeclampsia could be associated with the degree of maternal well-being of the participants. In other words, if the participants were affected with severe preeclampsia, the CRC is also diminished (compared to controls, *p*<0.01; **Figure 2**). Furthermore, if participants were affected with mild preeclampsia, the CRC is diminished compared to controls, *p*<0.05). Lower p-values could be associated with preeclampsia severity (compared to controls). However, no significant differences in MI and RMI (**Figures 2A, B**) were found between P and SP. It suggests that the CRC disruption may be similar between these two groups, regardless of the changes in vagal activity.

Lower values of MI and RMI may indicate decreased CRC. In contrast, higher values may imply an increased CRC. Therefore, the lowest CRC was manifested in the participants with severe preeclampsia. Other studies have already reported an altered CRC during different physiopathological states, such as depression (44), schizophrenia (58), apneas of prematurity, sudden infant death syndrome, obstructive sleep apnea, and Rett syndrome (7). These approaches have concluded probably exists a relationship between a diminished CRC and physiopathological processes, which typically change vagal nerve activity. It is related to dysautonomia in the regulatory functions of the cardiac and respiratory systems.

**Figure 5:**
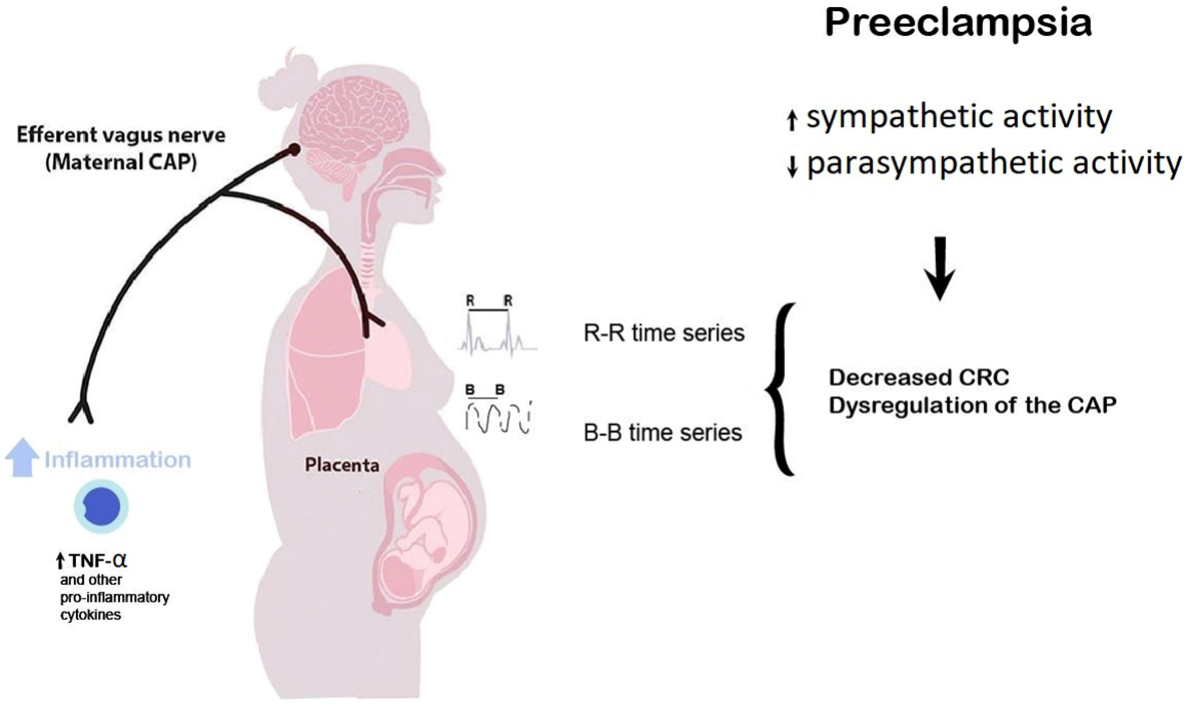
Preeclampsia may be considered a suitable model of high sympathetic and low parasympathetic activity, reduced heart rate complexity, and exacerbated inflammation, probably mediated by an increased tumor necrosis factor-α (TNF-α) and other pro-inflammatory factors cytokines. The potential dysregulation in the maternal cholinergic anti-inflammatory pathway (CAP) may be reflected as a decreased cardiorespiratory coupling (CRC) with concomitant vagal withdrawal. The linear and nonlinear analysis of the maternal beat-to-beat (RR) and breath-to-breath (BB) time series allows for noninvasively quantifying the efferent vagus nerve activity (related to the CAP) extracted from cardiorespiratory dynamics.

The manifestation of high CRC creates synergies that promote healthy physiology. Consequently, and following the previous paragraph, a reduction in such coupling may suggest a decrease in the efferent vagal activity of the autonomic network, resulting in potential autonomic dysautonomia in the maternal organism due to parasympathetic control decrease (7). The diminished vagal modulation produced by preeclampsia may decrease the endothelial vasodilator molecules decrease (endothelial dysfunction), such as prostacyclin (PGI2), nitric oxide (NO, l-arginine derivative), and endothelium-derived hyperpolarizing factor, whose lack during pregnancy would generate a greater hypertensive crisis, which is typical of the preeclamptic state (59). According to Gerritsen and Band, it is possible to directly stimulate the vagus nerve by adopting a low respiration rate. Increased vagal activity is associated with higher HRV and CRC, potentially activating the cholinergic anti-inflammatory pathway or CAP (8). Thus, preeclamptic women may manifest a dysregulation of the CAP mediated mainly by tumor necrosis factor-α (TNF-α) and other pro-inflammatory cytokines (60) and decreased CRC (**Figure 5**).

We believe that utilizing multivariate physiological signals, such as CRC, for analysis consistently represents a novel method to investigate the well-being of women with preeclampsia. According to the results of this study, this approach could provide a supplementary means to monitor and assess preeclamptic pathology, as well as its severity. These clinical implications hold significant relevance for making decisions such as determining the need for an earlier delivery or continued administration of indicated medication. Thus, it is important to recognize the significance of diagnosing this disease objectively and accurately. Our future work will involve the development of a real-time monitoring device for performing CRC analysis. We will also incorporate machine learning models to aid in decision-making as patients are categorized with or without preeclampsia.

The intricate interplay between cardiorespiratory coupling and vagal response in mild and severe preeclampsia can account for the variations observed in linear and nonlinear indices. Preeclampsia and its severe form may be linked to disrupted cardiorespiratory coupling and diminished vagal activity. MI and RMI quantify the relationship between RR and BB time series, whereas logRSA, mean PRQ, and nPermEn_PRQ assess different facets of cardiorespiratory dynamics encompassing vagal activity, linear and nonlinear characteristics, and complexity. These indices offer complementary insights into CRC and its underlying mechanisms, and the diverse statistical differences detected in this study may be associated with the specific aspects of CRC that they can capture.

The logRSA index, mPRQ, and nPermEn_PRQ were the only indices that revealed significant differences between the P and SP groups, suggesting distinct alterations in vagal and cardiorespiratory interactions in preeclampsia. Specifically, the logRSA index may offer greater efficiency in quantifying the respiratory-related component and vagal tone compared to other traditional measures of HRV (37, 61). Furthermore, logRSA provides enhanced robustness compared to frequency-domain methods, enabling higher time-resolution analysis (37, 61). This robustness is critical as it helps protect the results from potential disruptions caused by movement artifacts and ectopic heartbeats. On the other hand, both linear features of PRQ fluctuations, such as mPRQ, and nonlinear features, like entropy, have demonstrated sensitivity to changes in the dynamic behavior of CRC, such as body posture and breathing patterns (22), as well as the ability to identify respiratory changes due to induced relaxation and potential parasympathetic activation (62). Therefore, this set of indices can likely identify subtle changes in CRC activity between the P and SP groups compared to the rest of the indices.

## 5 Limitations

When used in time series analysis, mutual information presents certain limitations. Due to its symmetric nature, it does not offer insights into whether variable X influences variable Y or vice versa. As a result, the direction of information flow remains undisclosed (63). Like other information theory measures, the mutual information method is sensitive to noise and artifacts (64). Future studies will employ alternative approaches such as bivariate phase-rectified signal averaging (65) or joint symbolic dynamics (66) to tackle this limitation to measure causality between the maternal beat-to-beat and breath-to-breath time series. Additional limitations imply the lack of measures of mediators of inflammation markers in all the participants. However, authors have considered using vagally-mediated HRV as relevant indices to indirectly estimate the cholinergic anti-inflammatory pathway activity (67, 68).

Due to global recommendations advocating for labor induction before 37 weeks of gestation in women diagnosed with preeclampsia (69), most preeclamptic participants (90%) had medical indications for undergoing C-section surgery. This specific clinical indication generated significant differences in the mean values of gestation age among groups (**Table 1**), which could have influenced our CRC results. Nonetheless, all the participants in the three groups were in the third trimester of pregnancy (> 36 weeks of pregnancy), and the main changes could be attributed to the preeclamptic condition (51, 52).

We conducted a supplementary analysis using multiple regression (data not shown) to explore the potential impact of gestational age on our findings. However, we did not observe any linear relationship between the variables under investigation and gestational age.

Finally, all the participants were diagnosed with moderate or severe preeclampsia based on standardized clinical criteria in the present study. It is important to note that none of the participants had received medication to treat symptoms associated with preeclampsia during pregnancy until they were admitted to the Emergency and Obstetrical Surgery Department of the Maternal-Perinatal Hospital “Mónica Pretelini Sáenz”. Upon admission, the medication (Nifedipine 30 mg and Metoclopramide 10mg) was administered within a 12-hour to ensure the patient’s clinical stability. This approach was necessary before proceeding with the physiological recordings and study procedures. Since the medication administration was consistent for all participants in the preeclampsia groups, we believe that the potential influence of medication on the study results has been minimized. The uniformity in medication use across the groups helps ensure that any observed differences in cardiac vagal modulation and cardiorespiratory coupling are more likely to be associated with the underlying condition of preeclampsia rather than medication effects.

## 6 Conclusion

The present study explored the autonomic changes in cardiorespiratory coupling between parturient normotensive control women and preeclamptic women with mild and severe preeclampsia utilizing nonlinear methods based on information theory (mutual information or MI, Rényi’s mutual information or RMI and the analysis of novel index of Pulse-Respiration Quotient or PRQ). Our findings suggest that women with severe and mild preeclampsia exhibit disrupted cardiorespiratory coupling compared to normotensive women. Lower values of MI and RMI indicated decreased cardiorespiratory coupling, particularly pronounced in severe preeclampsia. Furthermore, decreased vagal activity, as indicated by lower logRSA values, was also observed in severe preeclampsia. The variation in vagal activity observed between women with mild preeclampsia and those with severe preeclampsia could indicate a subtle decrease in vagal activity as the disease progresses.

Additionally, severe preeclampsia was associated with less complex cardiorespiratory dynamics, reflected by lower values of normalized permutation entropy of the PRQ time series. These novel observations highlight potential autonomic dysregulation in preeclampsia, which may contribute to endothelial dysfunction and hypertensive crises. The analysis of cardiorespiratory coupling could be considered a promising tool to provide additional clinical prognostic information, detect autonomic changes of vagal activity in women with preeclampsia, or provide complementary indicators of well-being to other conventional techniques used in the clinical field. The comprehensive analysis of CRC using nonlinear methods and PRQ analysis enhances our understanding of the pathophysiology of preeclampsia and its implications for clinical management.

## Data availability statement

The raw data supporting the conclusions of this article will be made available by the authors, without undue reservation.

## Ethics statement

The ethics committee reviewed and approved the studies involving human participants from the “Monica Pretelini Sáenz” Maternal-Perinatal Hospital in Toluca de Lerdo, Mexico State, Mexico (reference number: 2021-03-719). The patients/participants provided written informed consent to participate in this study. The studies were conducted in accordance with the local legislation and institutional requirements. Written informed consent was obtained from the individual(s) for the publication of any potentially identifiable images or data included in this article.

## Author contributions

Conceptualization, JJR-L. Methodology, EYP-C and RGC-J. Software, EYP-C, JJR-L, RGC-J, CIL-R, and HM-Z. Validation, MAP-C. Formal analysis, JJRL-L, and EYP-C. Data curation, JJR-L, and EYP-C. Writing—original draft preparation, YM-P, JJR-L, MAP-G. Writing—review and editing, YM-P, HM-Z, CIL-R, MAP-C, MAP-G and E-N. Supervision, JJR-L. Project administration, JJR-L, and HM-Z. Funding acquisition, YM-P. All authors contributed to the article and approved the submitted version.
